# Randomized controlled trial of an oral gastrin receptor antagonist for the treatment of postmenopausal osteoporosis

**DOI:** 10.1093/jbmr/zjaf165

**Published:** 2025-11-08

**Authors:** Marian Schini, Fatma Gossiel, Margaret A Paggiosi, Sara L Hilditch, Stuart More, Irvin Modlin, Richard Eastell

**Affiliations:** University of Sheffield, Sheffield S10 2TN, United Kingdom; University of Sheffield, Sheffield S10 2TN, United Kingdom; University of Sheffield, Sheffield S10 2TN, United Kingdom; University of Sheffield, Sheffield S10 2TN, United Kingdom; University of Cape Town, Cape Town 7700, South Africa; Keewaydin Consulting Inc, 650 Park Ave, New York, USA; University of Sheffield, Sheffield S10 2TN, United Kingdom

**Keywords:** osteoporosis, treatment, netazepide, gastrin receptor antagonist, bone turnover markers, clinical trial

## Abstract

High gastrin levels may help to explain the association between several conditions and osteoporosis, such as pernicious anemia, the use of proton pump inhibitors, and atrophic gastritis. This study aimed to determine whether administering a gastrin receptor antagonist (GRA) to older women would lower their bone turnover markers (BTM) and, therefore, be a suitable preventive measure for osteoporosis. We conducted a randomized, double-blind, placebo-controlled clinical trial to assess the efficacy, safety, and tolerability of an oral GRA (netazepide) 100 mg administered daily for 90 d in postmenopausal women. Our primary endpoint was the change in the BTM plasma CTX (automated immunoassay analyzer) at days 0, 7, 28, 56, and 90. We also measured other BTMs, and gastrin and group I pepsinogens (ELISA assays). We studied the effect of the drug on the log-transformed baseline scaled ratio for BTM and gastric markers using mixed-model ANOVA for the fixed effects of treatment, time, and the treatment-by-time interaction, with the baseline value included as a covariate. We studied 99 women, with a mean age of 60 yr and bone mineral density (BMD) T-scores for the spine and total hip (TH) of −0.96 and −0.09, respectively. We found that gastrin increased by 90% in response to GRA as early as 7 d (*p*-value for treatment: .0008), and group I pepsinogens decreased by 15% as early as 7 d (*p*-value: .0002). There was no significant change in plasma CTX. A high percentage of women (81/99) completed the study, and the GRA was well tolerated. Gastrin receptor antagonist had the expected effects on the gastric markers with an increase in gastrin and a decrease in group I pepsinogens. However, the absence of any change in the bone resorption marker plasma CTX was a bit surprising. Based on this study, it appears that short-term gastrin receptor antagonism is unlikely to be a successful strategy in the prevention of osteoporosis. However, this is a preliminary exploration of a novel hypothesis and larger studies might be needed.

## Introduction

Osteoporosis is a very common condition that can cause fragility fractures. Postmenopausal women are especially at risk due to estrogen deficiency. Several medications are used to treat osteoporosis, but there are limitations to their use; these medications can also exhibit adverse events (AEs), which can restrict their use.^1^ Other biological factors can contribute to the development of osteoporosis and could be considered for use as potential treatment targets.

Hypergastrinemia is a clinical entity that can be identified in up to 10% of older women in the general population.^2^ Atrophic gastritis is found in 27.1% of autopsies, with prevalence and severity increasing with age.^3^ Pernicious anemia (PA) is an autoimmune disease characterized by antibodies against the parietal cells leading to vitamin B12 deficiency and achlorhydria. Pernicious anemia can cause hypergastrinemia^4^ and has been associated with detrimental effects on bone health. Subjects with PA had an increase in the risk of fractures compared to controls (1.9-fold increase in proximal femur, 1.8-fold increase in vertebral, and 2.9-fold increase in distal forearm fractures).^5^ In another study, the risk for hip fractures in people with PA was reported as 1.74 higher than controls (HR: 1.74, 95% CI: 1.45-2.08).^4^ A decrease in BMD at the lumbar spine (LS) up to 16% was seen in 1 study when PA patients with PA were compared with healthy controls.^6^ Furthermore, separate studies have reported lower mean BMD values at the hip (men) and spine^7^ in people with vitamin B12 deficiency (<148 pmol/L).^8^

The consideration that increased levels of gastrin associated with atrophic gastritis might lead to alterations in BMD is supported by the reports of long duration proton pump inhibitor (PPI) therapy (associated with profound acid suppression and sustained hypergastrinemia) and bone disease, in particular osteoporosis.^9^ Previous studies have shown that PPI use can cause similar findings to PA (ie, low B12, hypergastrinemia, and calcium malabsorption).^10^^,^^11^ The use of PPI is commonplace and according to a recent study, more than 20% of the population takes PPIs worldwide. The majority of individuals on long-term PPI therapy are older adults, but young- to middle-aged adults are also substantial users.^12^

There have been contradictory data describing the association between PPI use and fracture risk. A systematic review performed in 2012, collected data from 14 observational studies performed between 1980 and 2011. Eight of these studies identified an increase in the risk of hip fractures (20%-62%) and 5 in the risk of vertebral fractures (40%-60%). Three studies showed that if PPIs are discontinued from one month up to 1 yr, the fracture risk decreases. Three studies assessed BMD but did not find consistent results. Some studies showed that higher doses of PPIs might be associated with higher fracture risk.^13^ A more recent review of 18 observational studies (published in the 5 yr up to 2019) concluded that PPIs were positively associated with elevated fracture risk in multiple studies (*n* = 14). It is noteworthy, however, that some studies reported no significant relationship (*n* = 4), perhaps suggesting the association of other variables in certain populations.^14^

Given these observations, we considered that a gastrin inhibitor might be beneficial in the treatment of osteoporosis. Netazepide (YF476), a selective CCK2R antagonist (gastrin receptor antagonist, GRA), has been under development since 1997.^15^ The majority of animal studies have focused on its use as an inhibitor of gastrin-mediated effects in the stomach. To date, its clinical efficacy has been mostly confined to its ability to inhibit the growth of rodent and human gastric carcinoid tumors. Several studies have been performed with patients taking the GRA (25-100 mg) by mouth for up to 12 wk^16–18^ with a study extending by a further 52 wk.^19^ All studies identified a significant decrease in tumor size. Netazepide data on osteoporosis are limited and are only available in animal models. Knockout (KO) mice (H-K-ATPase beta subunit knockout) were found to exhibit hypergastrinemia and lower BMD compared to WT mice.^20^^,^^21^ Treatment with netazepide, induced a higher cortical thickness, cortical area fraction, trabecular thickness, and trabecular volumetric BMD (seen by micro-CT).^20^

With this as our background concept, we considered the clinical observation that older women are more likely to exhibit a low acid state (atrophic gastritis with associated hypergastrinemia). The consequences of the elevated levels of this gastric hormone are supported by the link between PA or PPI and fracture risk. High gastrin might mediate this propensity to fracture, although, to our knowledge, gastrin receptors have not been identified in bone.^20^ Given these findings, we hypothesize that administration of an oral GRA might reduce the high bone turnover observed in the postmenopausal state. To the best of our knowledge, the effect of netazepide in osteoporosis has not previously been explored in humans.

## Methods

OSTEOGRA is a phase IIb, 1:1 randomized, double-blind, parallel, placebo-controlled clinical trial to assess the efficacy, safety, and tolerability of netazepide 100 mg daily administered for 90 d in postmenopausal women. The study was conducted in the Groote Schuur Hospital, Cape Town, South Africa. The study was registered with the South African National Clinical Trials Registry (SANCTR) database (https://www.sahpra.org.za/, number = DOH-27-0519-6154) and the Pan African Clinical Trial Registry (PACTR) database (https://www.pactr.samrc.ac.za, number PACTR202407466904092). The primary objective of this study was to examine the effect of the GRA on plasma cross-linked carboxy-terminal telopeptide of type I collagen (CTX) levels compared to placebo in postmenopausal women. We also examined the effects on the bone formation marker, PINP.

Eligibility was defined as women that met the following criteria: (1) were >5 yr postmenopausal or had undergone an oophorectomy, (2) had FSH levels of >40 IU/L at the screening visit, (3) showed no clinical or biochemical evidence of secondary causes of bone loss, and (4) were not taking medications known to affect bone metabolism. Our exclusion criteria were based on (1) a confirmed diagnosis of a disease known to cause secondary bone loss (including diabetes mellitus, thyroid dysfunction, coeliac disease, pancreatic exocrine insufficiency, epilepsy, and hyperparathyroidism); (2) sustained a bone fracture in the previous year; and (3) undergone orthopedic surgery in the previous year.

Fasting blood samples were drawn by a trained phlebotomist. All samples were aliquoted and then stored within −80° C freezers at the study site. Study-specific safety testing of participant samples was performed by a local accredited research laboratory (National Health Laboratory Services, Groote Schuur Hospital). Following the last patient, last visit (LPLV), all samples were shipped to the Division of Clinical Medicine, School of Medicine and Population Health, University of Sheffield, Sheffield, UK, where bone turnover marker measurements were performed. Total serum procollagen type I N-propeptide (PINP), osteocalcin (OC), and plasma C-telopeptide of type I collagen (CTX) were measured using the Cobas e411 automated immunoassay (Roche Diagnostics). Tartrate-resistant acid phosphatase (TRAP5b) and bone alkaline phosphatase (ALP) were measured using IDS-iSYS automated immunoassays (Immunodiagnostic Systems). N-terminal telopeptide (NTX) was measured using the Ortho Eci automated immunoassay (Ortho Clinical Diagnostics). N-terminal telopeptide was expressed as a ratio to creatinine. Type 1 pepsinogen (PG1) was measured using a manual sandwich ELISA (Abcam Limited). Gastrin was measured using Siemens Immulite 2000, parathyroid hormone (PTH) using Roche Cobas e801, 25OHD using Abbott Alinity I and chromogranin A(CgA) using Brahms Kryptor (Thermo Fisher). Results that were below the measuring range of the assay were reported as the limit of detection of the assay. We used a value of 0.5 × the limit of detection for any low values. Results that were “extreme outliers” (eg, above the detection limit of the assay) were reassessed for further investigation after review of the participant’s clinical notes. If a recognized cause of high bone turnover was noted, the results were included in the intention-to-treat (ITT) analysis but excluded from the per-protocol analysis. The precision of each test was determined by calculating the inter-assay coefficient of variation.

A single dose of vitamin D (100 000 IU) was administered to study participants during the screening visit if their 25OHD <20 ng/mL. Following this, participants received 90 d of: ARM 1—IMP: netazepide 100 mg (4 × 25 mg GRA capsules—YF476 taken orally in the morning after breakfast) daily or ARM 2—matching placebo (4 × capsules taken orally in the morning after breakfast) daily. The study medication was provided by the manufacturer, Biophore. No toxicity was anticipated as Phase II studies had demonstrated no AEs in patients (treated with netazepide for 52 wk). If a participant in this study developed a serious adverse event (SAE), the dose was reduced to 50 mg, that is, 2 capsules daily.

To minimize potential bias, participants were randomized to receive either netazepide or placebo in a blinded manner using a computer-generated algorithm (random number generator) within STATA. A randomization list was produced and stored securely in the University of Cape Town pharmacy. Once screening and enrolment had been completed successfully by the study team, pharmacy staff randomized the participant to receive either netazepide or placebo sequentially as indicated on the randomization list.

Compliance was assessed at each follow-up visit via tablet counts. Any discrepancies between the actual and expected amount of returned study medication were discussed with the participant and documented in the source notes. If poor compliance was noted, the participant was counseled.

A minimum of at least 41 participants per arm were required to have 90% power and to consider a significant result to be a *p*-value of .05 (2-tailed). This assumed a clinically significant difference in the bone resorption marker CTX of >25% after treatment with the GRA. This figure was based on a mean pretreatment level of 0.68 ng/mL (range: 0.47-0.93). Drugs that reduce the risk of fracture such as raloxifene^22^ result in a change in CTX of this magnitude.^23^

The ITT analysis included all the data from all individuals recruited and enrolled on the OSTEO-GRA study, and then randomized to receive either GRA or placebo, up to study completion or the point of their withdrawal (ie, the full data analysis set). All enrolled and randomized subjects were included in this primary analysis to ensure an unbiased estimate of the treatment effect. The AEs (ITT analysis only) were reported using the Medical Dictionary for Regulatory Activities Terminology (MedDRA) classification (https://www.meddra.org/).

The per-protocol analysis included the data from all the individuals recruited and enrolled on the OSTEO-GRA study and then randomized to receive either GRA or placebo. However, only data for participants who fulfilled all of the following criteria were included: (1) completed all study visits and (2) ≥80% compliant with their assigned study medication. Any participants found to be subsequently ineligible (eg, extreme outliers which may have indicated possible underlying disease) were excluded. We followed the iterative exclusion of outliers’ process of sequentially identifying and removing extreme data points, recalculating distribution thresholds after each step, until no further outliers remain, or a predefined criterion was met. Measurement were considered extreme outliers if they were three times the interquartile range (IQR) or more above or below the IQR, either from raw data at any time point or percentage change at any time point were considered extreme outliers. If one value was found to be an outlier, then that entire participant was excluded from the study.

Statistical analyses of the change in BTMs were based on previously described approaches.^23^^,^^24^ As the percentage changes in BTMs were predicted to be negative, we calculated baseline scaled ratios (BSR) (as follows) for each BTM. These BSRs were used when assessing the statistical significance of change. We calculated the percentage change in BTM by subtracting each on-treatment BTM from the baseline BTM, dividing this difference by the baseline BTM, and then multiplying this fraction by 100 to get a percentage. We calculated the BSR of BTMs (BSR BTM) by dividing each on-treatment BTM by the baseline BTM. We checked for normality and log transformed where necessary. The analysis of change in BTMs were estimated using a repeated measures mixed model with change from baseline (on a log scale as described above) as the outcome, treatment, and timepoint as factors together with a treatment by timepoint interaction, subject as a random effect and baseline as a covariate. The model was used to estimate the change from baseline to each timepoint (week 1, week 4, week 8, and week 12).

All analyses were performed using: GraphPad Prism 10 for Windows 64-bit (Version 10.3.1 (509), GraphPad Software LLC, August 16, 2024, GraphPad Prism) and RStudio (Version 2024.09.0+375), Posit, 23 August 2024, RStudio). All analyses were carried out with a 2-sided 5% significance level. A *p*-value <.05 for the primary endpoint indicated statistical significance. No adjustment was made for multiplicity in the other endpoints, so these should be interpreted with caution.

## Results

Eighteen patients did not complete the study. The study started in 2020 and completed in 2023. Some patients did not complete the study because the sponsor decided to stop the study due to the COVID pandemic (*n* = 8). Two stopped due to poor compliance, 1 due to low tolerability, 1 due to incorrect dosing schedule, 1 due to difficult venepuncture, 2 due to concomitant medications, which could affect bone metabolism (furosemide and antiretrovirals), and 3 due to abnormal investigations. No participant required a dose reduction. Forty-four patients had high-dose vitamin D before starting (27 from the placebo group and 17 from the GRA group). Race data was not available, as this is considered to be a sensitive information by the government of South Africa.

### Intention-to-treat analysis

**Figure 1:**
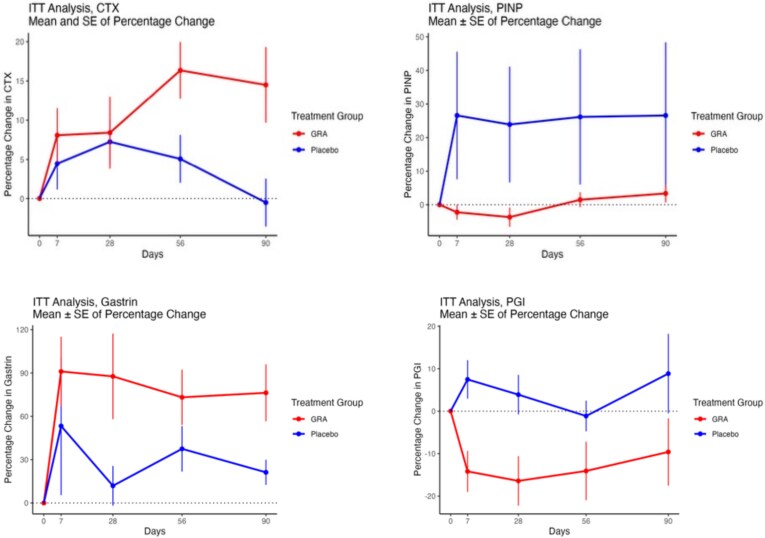
Plots of percentage change (mean ± SE). CTX, C-telopeptide of type I collagen; ITT, intention to treat; PINP, procollagen type I N-terminal propeptide; PGI, group I pepsinogen.

A total of 99 women were recruited with a mean age of 60 yr and BMD T-scores for the spine and TH of −0.96 and −0.09, respectively (Table 1). Gastrin increased by 90% in response to GRA as early as 7 d (*p*-value for treatment .0008), and group I pepsinogens decreased by 15% as early as 7 d (*p*-value for treatment .0002) (Figure 1 and Table 2). There was no significant change in plasma CTX. PINP showed a significant treatment^*^time interaction (*p*-value .0236) but subsequent pairwise comparisons did not show any significant results. For bone ALP, there was a statistical difference at day 7 (*p* = .0407).

**Table 1:** Baseline characteristics, ITT analysis, and median (IQR).

**Variable**	**GRA**	**Placebo**
	**(*N* = 49)**	**(*N* = 50)**
**Age, (yr)**	59.9 (54.3-63.7)	59.9 (55.9-65.7)
**Weight, (kg)**	84.8 (73.3-97.0)	73.4 (68.2-88.5)
**Height, (m)**	1.60 (1.57-1.65)	1.60 (1.56-1.62)
**BMI, (kg/m** ^**2**^ **)**	32.0 (27.3-38.1)	28.6 (25.9-34.3)
**LS, BMD T-score**	−1.00 (−1.90 to 0.05)	−1.20 (−1.92 to −0.50)
**TH, BMD T-score**	−0.10 (−0.70 to 0.70)	−0.15 (−1.10 to 0.60)
**Number (%) with previous fractures**	7 (14%)	9 (18%)
**CTX, (ng/mL)**	0.469 (0.352-0.613)	0.494 (0.373-0.593)
**PINP, (ng/mL)**	70.1 (56.5-90.2)	63.5 (45.9-83.0)
**Gastrin, (pg/mL)**	28.0 (16.0-49.5)	29.0 (17.5-41.5)
**PGI, (ng/mL)**	50.3 (39.0-104.5)	91.6 (64.5-133.9)
**25OHD, (ng/mL)**	22.0 (16.0-26.0)	19.0 (14.8-24.5)

Abbreviations: CTX, C-telopeptide of type I collagen; GRA, gastrin receptor antagonist; PGI, type I pepsinogen; PINP, procollagen type I n-propeptide.

**Table 2:** Outcomes of the mixed effects regression modeling (2-way ANOVA).

**Mixed model analysis (ITT)**	**ln baseline**	**Treatment**	**Time**	Treatment^*^Time interaction
**Bone resorption markers**				
** CTX (primary endpoint)**	**<0.0001**	0.2610	0.4785	0.2189
** NTX**	** 0.02916**	0.34008	0.80751	0.43985
** TRAP5b**	**<0.0001**	0.5772	0.3626	0.5253
**Bone formation markers**				
** PINP**	**<0.0001**	0.7337	0.3461	**0.0236**
** Osteocalcin**	** 0.002485**	0.515320	0.238957	0.680282
** Bone ALP**	**<0.0001**	0.360467	0.488751	**0.007669**
**Gastric markers**				
** Gastrin**	**<0.0001**	**0.0008124**	0.5085762	0.8227678
** PGI**	** 0.0026550**	**0.0001533**	0.4154725	0.8709743

Abbreviations: ALP, alkaline phosphatase; CTX, C-telopeptide of type I collagen; ITT, intention to treat; NTX, N-terminal telopeptide; PINP, procollagen type I N-terminal propeptide; PGI, group I pepsinogen; TRAP5b: tartrate-resistant acid phosphatase 5b.

### Per-protocol analysis

**Figure 2:**
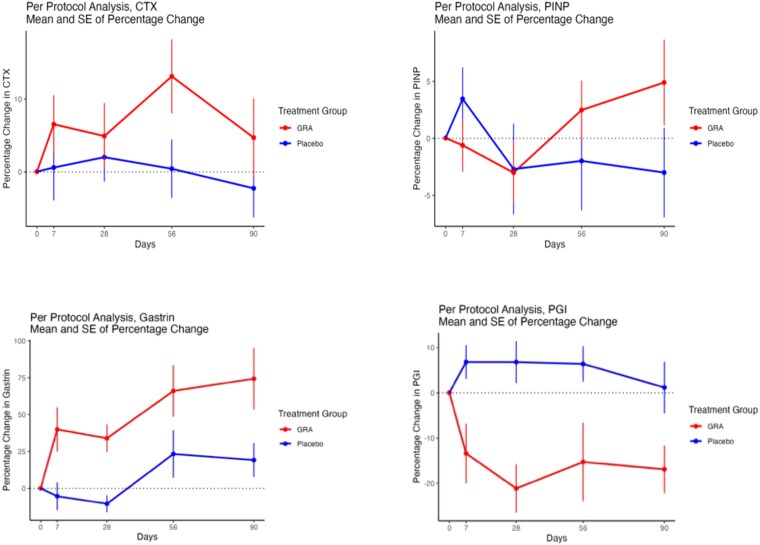
Plots of percentage change (mean ± SE), per-protocol analysis. CTX, C-telopeptide of type I collagen; PINP, procollagen type I N-terminal propeptide; PGI, group I pepsinogen.

The baseline characteristics of the per-protocol analysis were similar to the ITT (data not shown). There were 25 participants in each group for the primary outcome analysis (CTX). Once again, the gastric markers responded as expected in response to GRA (*p*-value for treatment .003 for gastrin and .03 for group I pepsinogens (PGI)) (Figure 2 and Table 3). There was no significant change in plasma CTX or PINP. Pairwise comparisons were performed for bone ALP and showed a statistical difference at day 90 (*p* = .0028).

**Table 3:** Mixed effects regression modeling (2-way ANOVA) for the per-protocol analysis.

**Mixed model analysis (per-protocol analysis)**	**ln baseline**	**Treatment**	**Time**	Treatment^*^Time interaction
**Bone resorption markers**				
** CTX (primary endpoint)**	**0.00253**	0.06960	0.46500	0.57980
** NTX**	**0.000242**	0.743386	0.857301	0.538316
** TRAP5b**	**0.006665**	0.245364	0.310063	0.579927
**Bone formation markers**				
** PINP**	**0.006448**	0.110560	0.299817	0.131599
** Osteocalcin**	**0.0193**	0.3985	0.1048	0.3430
** Bone ALP**	0.13147	0.20604	0.65004	**0.01379**
**Gastric markers**				
** Gastrin**	**0.0027068**	** 0.0003806**	0.0209827	0.9951252
** PGI**	**0.02742**	**<0.0001**	0.51239	0.35878

Abbreviations: ALP, alkaline phosphatase; CTX, C-telopeptide of type I collagen; NTX, N-terminal telopeptide; PINP, procollagen type I N-terminal propeptide; PGI, group I pepsinogen; TRAP5b, tartrate-resistant acid phosphatase 5b.

### Adverse events

There were 31 participants with AEs from the GRA group and 33 from the placebo (Table 4). One from the GRA group was reported as having a SAE. This was an abscess of the left arm that required surgical drainage. They were 6 grade 3 AE reported in 4 patients [increased: hemoglobulin A1c (HbA1c), gastrin in 2 participants, chromogranin A, alanine aminotransferase (ALT), and aspartate aminotransferase (AST)] (Table 4).

**Table 4:** Number participants with adverse events per treatment group categorized using Medical Dictionary for Regulatory Activities Terminology (MedDRA) classification.

**Adverse event per MeDRA system organ class (SOC)**	**GRA (*n* = 49)**		**Placebo (*n* = 50)**	
	**Number**	**%**	**Number**	**%**
**Blood and lymphatic system disorders**				
** Anemia**	1	2	2	4
**Cardiac disorders**				
** Chest pain**	1	2	1	2
**Ear and labyrinth disorders**				
** Vertigo**	0	0	1	2
** Acute otitis media with effusion**	1	2	0	0
**Endocrine disorders**				
** Diabetes**	3	6	0	0
**Eye disorders**				
** Conjunctivitis**	1	2	0	0
**Gastrointestinal disorders**				
** Abdominal pain** ^**a**^	0	0	3	6
** Constipation**	0	0	1	2
** Diarrhea** ^**a**^	2	4	1	2
** Dyspepsia**	2	4	0	0
** Dysphagia**	0	0	1	2
** Epigastric pain**	0	0	1	2
** Gastroenteritis**	3	6	1	2
** Heartburn**	1	2	1	2
** Loose stools**	3	6	1	2
** Nausea** ^**a**^	2	4	2	4
** Toothache**	0	0	1	2
** Vomiting**	1	2	0	0
**General disorders and administration site conditions**				
** Fatigue** ^**a**^	1	2	1	2
** Flu-like symptoms**	3	6	2	4
** Flush hot**	1	2	0	0
** Lethargy**	1	2	1	2
** Pitting edema**	0	0	1	2
**Infections and infestations**				
** Abscess**	1	2	0	0
** Ringworm**	0	0	1	2
** Upper respiratory tract infection**	2	4	3	6
** Urinary tract infection**	1	2	1	2
**Injury, poisoning and procedural complications**				
** Fracture** ^**b**^	0	0	1	2
**Investigations**				
** Alanine aminotransferase increased**	1	2	0	0
** Aspartate aminotransferase increase**	1	2	0	0
** AST increased**	1	2	0	0
** Cholesterol levels raised**	2	4	1	2
** CK increased**	1	2	0	0
** Gastrin increased** ^**a**^	3	6	1	2
** Glucose increased**	1	2	1	2
** Hyperkalemia**	0	0	1	2
** Platelets increased**	0	0	1	2
** Potassium increased**	1	2	1	2
** TSH decrease**	0	0	3	6
** HbA1C increased**	5	10	4	8
** Blood chromogranin A increased**	2	4	0	0
**Metabolism and nutrition disorders**				
** Vitamin D deficiency** ^**b**^	1	2	0	0
**Musculoskeletal and connective tissue disorders**				
** Back pain**	4	8	1	2
** Joint pain**	3	6	0	0
** Knee pain**	1	2	0	0
** Muscle spasm**	0	0	4	8
** Musculoskeletal pain**	0	0	1	2
** Pain in hip**	1	2	0	0
** Shoulder pain**	1	2	0	0
** Shin splints**	0	0	1	2
**Nervous system disorders**				
** Cerebrovascular accident**	1	2	0	0
** Headache** ^**a**^	8	16	6	12
** Tremor**	0	0	1	2

(*Continued*)

**Table 4:** Continued

**Adverse event per MeDRA system organ class (SOC)**	**GRA (*n* = 49)**		**Placebo (*n* = 50)**	
	**Number**	**%**	**Number**	**%**
**Psychiatric disorders**				
** Anxiety**	0	0	1	2
**Renal and urinary disorders**				
** Dysuria**	0	0	1	2
**Reproductive system and breast disorders**				
** Vaginal bleeding**	1	2	0	0
**Respiratory, thoracic and mediastinal disorders**				
** Cough**	2	4	1	2
** Epistaxis**	1	2	0	0
**Skin and subcutaneous tissue disorders**				
** Nodule skin**	0	0	1	2
** Pruritus**	1	2	0	0
** Rash**	1	2	0	0
**Vascular disorders**				
** Hypertension**	3	6	0	0

a AEs reported in previous studies with gastrn receptor antagonist (GRA).

b AEs of special interest to bone.

We identified some adverse effects in assessment of the laboratory measurements. There was a significant interaction between treatment and time for hemoglobin, hematocrit, and red blood cell count. This comprised a slight mean reduction (<5%) at day 56 for hemoglobin and hematocrit (not significant for red blood cells). This was reversed by 90 d despite continuous treatment. There were 2 influential points (with decreases of 11% and 13%), but these did not develop anemia (Hb < 11.6 mg/dL) and returned to baseline by day 90. We noted that 1 woman in the GRA group had a temporary increase in ALT and AST. This individual also exhibited elevations in alkaline phosphatase, which did not exceed the upper limit of normal.

There were 2 participants with increased platelets: 1 in the GRA group and 1 in the placebo group; both changes were transient. One woman in the placebo group had a significant but transient decrease in serum magnesium for which no cause was noted, and the low level was reversible. Two women in the GRA group had increases in thyroid stimulating hormone (TSH), but these reversed without any specific treatment.

## Discussion

We conducted a randomized, double-blind, placebo-controlled clinical trial to assess the efficacy, safety, and tolerability of an oral GRA (netazepide or GRA) 100 mg administered daily for 90 d in postmenopausal women. Gastrin receptor antagonist had the expected effects on the gastric biomarkers with an increase in gastrin and decreases in PGI and CgA. It did not affect BTMs. Gastrin receptor antagonist did not cause any higher rate of AEs than the placebo.

The lack of effect of GRA on BTM did not support our hypothesis. The results were the same for all subjects (ITT analysis) and for a subset that completed the study, were treatment compliant and had no other likely condition affecting bone (per-protocol analysis). There was 1 significant result in the efficacy analysis for both ITT and PP, namely the bone ALP, which increased in the placebo group but did not change in the GRA group. This is an unexpected change that outliers cannot explain. It likely occurred because of chance as it only affected the placebo group. All currently used agents used in the treatment of osteoporosis result in changes to these markers, so it is unlikely that the GRA would prevent osteoporosis.

We considered whether we had used the correct dose and whether the drug had been formulated correctly. The dose and duration of 100 mg daily netazepide for 3 mo is similar to previous trials, which had doses of up to 200 mg daily^25^ for up to 3 mo and were used for the treatment of gastric carcinoid. In healthy subjects, the effect of netazepide is to increase serum gastrin after 6 wk and decrease chromogranin A as in the present study.^26^ No study has measured PGI, but an increase in gastric pH results in lowering of PGI secretion by the chief cells of the stomach. Thus, netazepide would be expected to decrease PGI levels as in the present study. The highly significant changes in these gastric markers in the expected direction provides reasonable evidence to consider that netazepide was biologically effective. The dose was sufficient to affect the gastric markers; we cannot be certain that the dose was sufficient to alter bone turnover, but 3 mo has been shown to be adequate to identify the effects of all currently licensed drugs for osteoporosis.

The AE profile was similar between GRA and placebo. We paid particular attention to any fractures (but there was only one of these) and vitamin D deficiency (but there was only one of these). Previous studies had raised the possibility of other side effects with this class of drugs, including mild nausea, diarrhea, abdominal discomfort, transient headaches, and occasional fatigue. However, none of these were higher in the GRA group, and the present study of 99 women is significantly larger than previous studies. Prior studies are challenging to interpret for AEs, as they were often done in patients with diseases, such as chronic atrophic gastritis, and other drugs were co-administered (such as esomeprazole).^19^ Thus, the present study is the most extensive comparison of GRA against placebo in otherwise healthy people and should be of help in future studies, in providing reassurance about AEs.

We identified some abnormalities in the laboratory measurements. The changes in the hemoglobulin are unlikely to be clinically significant, because they were small and reversible. We noted one woman in the GRA group had significant increases in ALT and AST, markers of hepatocellular damage, at day 90. Still, these changes were transient and subsequently returned to normal by day 180 without specific treatment. We cannot ascertain whether these changes were netazepide-related. The alteration mentioned for magnesium, platelets, and TSH were uncommon, not related to symptoms, occurred in both the GRA and placebo group, and on remeasurement were reversed. They were unlikely to be significant.

To our knowledge, this is the first human study of bone health using an oral GRA. There was a large number of participants, with measurements of both bone resorption and formation markers but also gastric biomarkers. Group I pepsinogens results have never been reported. CTX was measured in plasma, as recommended. There are a few things to note. The power calculation mentioned that the expected CTX level would be 0.68 ng/mL, and it was 0.50. This lower-than-expected median CTX level may have been due to the characteristics of the population; the median BMI was around 30, the borderline between being overweight and obese, and obesity is associated with a lower CTX. However, this is only a slight difference and unlikely to be a cause of failure to respond. Diabetes is also known to cause low BTM but there were no diabetic people included in the study. The study could have included men, but they tend to have lower CTX than women.^27^ We can only generalize to women in South Africa. It could have included women taking PPIs or suffering from PA, as they would both have higher gastrin. However, both conditions are confounded by the presence of other diseases (peptic ulcer disease) or co-existent illnesses (eg, autoimmune disorders). Older women in the group are at the highest risk of osteoporosis and fragility fractures and so were considered to probably represent the most appropriate population. Lastly, only half of the subjects qualified for the per-protocol analysis, so this cannot really be considered a definitive assessment of the experimental question.

In conclusion, GRA had the expected effects on the gastric markers with an increase in gastrin and a decrease in group I pepsinogens. However, the absence of any change in the BTMs in 3 mo was unanticipated. Short-term gastrin receptor antagonism is unlikely to be a successful strategy in the prevention of osteoporosis, but, as this is a preliminary study, larger studies might be needed.
